# Evidence on Effectiveness of Upper Neck Irradiation Versus Whole Neck Irradiation as Elective Neck Irradiation in Node-Negative Nasopharyngeal Cancer: A Meta-Analysis

**DOI:** 10.1200/JGO.2016.006759

**Published:** 2016-11-30

**Authors:** Jayson L. Co, Michael Benedict A. Mejia, Janine Margarita R. Dizon

**Affiliations:** **Jayson L. Co** and **Michael Benedict A. Mejia**, Benavides Cancer Institute, University of Santo Tomas Hospital, Espana; and **Janine Margarita R. Dizon**, Center for Health Research and Movement Science, University of Santo Tomas, Manila, the Philippines.

## Abstract

**Purpose:**

Nasopharyngeal carcinoma (NPC) is a central tumor with a rich lymphatic network and a propensity for bilateral cervical lymph node metastasis. There is an orderly pattern of lymph node involvement in NPC. There is no current standard for prophylactic neck irradiation in node-negative or limited retropharyngeal (RP) node–positive NPC. This study aims to synthesize the current evidence on upper neck irradiation (UNI) versus whole neck irradiation (WNI) as prophylactic neck irradiation in node-negative or limited RP node–positive NPC.

**Materials and Methods:**

A search of relevant articles was done from 2000 to October 2015. Critical appraisal and meta-analysis of the eligible studies were undertaken to assess the effectiveness of UNI versus WNI as prophylactic neck irradiation in node-negative or limited involved RP node NPC.

**Results:**

Only one randomized controlled trial investigated the use of prophylactic UNI versus WNI and showed no confirmed nodal relapse in both arms. Pooled analysis of four retrospective studies showed no significant difference in nodal recurrence, whether in-field or out-of-field recurrence. There was also no significant difference in terms of 5-year distant metastasis–free and overall survival.

**Conclusion:**

In node-negative or limited RP node–positive NPC, the current evidence shows the possibility of treating only the upper neck (levels II, III, and VA) without compromising nodal control, distant metastasis, and overall survival. As a result of the scarcity of data, more randomized clinical trials are warranted in this subset of patients.

## INTRODUCTION

Nasopharyngeal carcinoma (NPC) is a prevalent malignancy in Asia, particularly southern China and Southeast Asian countries. The incidence of NPC in Asia is 1.4%, whereas in the Southeast Asian region, it is 5%.^1^ In the Philippines, one of the countries in the Southeast Asian region, local reports of NPC data revealed an incidence of 1.2 per 100,000.^2^ The treatment of NPC involves primary radiotherapy in early stages and concurrent chemoradiation followed by adjuvant chemotherapy for locally advanced disease.^3-5^

NPC is a central tumor with a rich lymphatic network and a propensity for bilateral cervical lymph node metastasis. Lymph node metastasis in NPC demonstrates an orderly pattern, and skip metastasis is rare. A prospective study of 271 patients with NPC showed orderly progression of metastasis through clinical palpation and recommended irradiation of one nodal level further from the involved lymph node level.^6^ Another study based on magnetic resonance imaging (MRI) showed that level II and the retropharyngeal (RP) lymph nodes were frequently involved, with orderly progression to levels III, V, and IV and the supraclavicular fossa, with only 0.5% of patients demonstrating skip metastasis.^7^ Limiting radiation fields to the upper neck for prophylactic neck radiation may decrease the dose received by the thyroid gland, carotid artery, lung apex, larynx, trachea, and soft tissues of the neck. Because of the propensity of NPC for bilateral lymph node metastasis, current protocols in NPC suggest irradiation of RP nodes; level II, III, IV, and V nodes; and the supraclavicular fossa regardless of nodal status.^8,9^ There has been controversy regarding and no current standard for prophylactic neck irradiation in node-negative NPC or only RP node–positive NPC. The aim of this article is to synthesize the current evidence regarding efficacy of upper neck irradiation (UNI) versus whole neck irradiation (WNI) as prophylactic neck radiation in node-negative or limited RP node–positive NPC.

## MATERIALS AND METHODS

Both published and unpublished English-language studies from 2000 to October 2015 were sought using the search terms “nasopharyngeal carcinoma,” “node negative,” and “neck radiation,” in MEDLINE Complete, CINAHL Plus, ProQuest Health and Medical Complete, Academic Search Complete, Biomedical Reference Collection Basic, and PubMed. Five studies were published in English, whereas for one study, only the abstract is in English. The reference lists of all identified publications (both included and excluded) were searched for additional studies. A Google Scholar search was also done. Two additional studies were included. Content experts were contacted to obtain additional references and unpublished trials. E-mails were also sent to try to obtain any unavailable data.

### Criteria for Considering Studies in This Review

#### Types of participants.

This review included studies of node-negative NPC determined by either computed tomography (CT) or MRI in accordance with the sixth edition of the American Joint Committee on Cancer (AJCC) Cancer Staging Manual (2002), published in cooperation with the International Union Against Cancer (AJCC/UICC). Studies that included NPC with RP nodal involvement were included, because this was considered as node-negative disease in the sixth edition of the AJCC/UICC staging system. Studies that used mere clinical palpation without imaging for the nodal staging were excluded. All studies had patients receiving radiotherapy to both primary and neck sites, with or without chemotherapy and with or without tumor boost.

#### Types of interventions.

The intervention included the use of either UNI or WNI as part of the elective neck irradiation (ENI). UNI included at least cervical levels II, III, and VA, whereas WNI included the addition of level IV and/or supraclavicular fields. The line of delineation was the cricoid cartilage. The dose received to this prophylactic area should be at least 50 Gy.

#### Types of outcome.

Studies with main outcome measures of nodal relapse, distant metastasis, and overall survival were included. Oncologic outcomes were measured using standardized reports of nodal relapse, distant metastasis, and overall survival. Nodal relapse was defined as absence of clinical or radiographic recurrence in the regional nodes. Distant metastasis–free survival was defined as the number of patients free of distant metastasis after 5 years, whereas overall survival was defined as the number of patients alive after 5 years from the date of treatment.

#### Types of studies.

This review included randomized controlled trials and retrospective comparative studies, which, because of the scarcity of evidence, were required to be able to obtain at least level 3 evidence. This is in consonance with the Oxford Centre for Evidence-Based Medicine, wherein retrospective cohorts are considered as level 3 evidence.^10^

#### Assessment of methodologic quality.

Two reviewers (J.L.C. and M.B.A.M.) conducted an independent critical appraisal of the eligible studies using a standardized critical appraisal form, the McMaster Critical Review Form–Quantitative Studies. There was no disagreement on the decision to include or exclude a study.

### Data Collection and Synthesis

Data were extracted independently by the two reviewers using a purpose-built Microsoft Excel spreadsheet (Microsoft, Redmond, WA). Data extraction included author, year, title, study design, sample size, study population, intervention, control, outcomes, and results. Statistical pooling was done for similar outcome using Review Manager Software 5.3 (The Nordic Cochrane Centre, The Cochrane Collaboration, Copenhagen, Denmark). Heterogeneity was assessed using χ^2^ analysis. In the presence of significant heterogeneity, a random-effects meta-analysis was used. Other data with dissimilar variables were collated using a narrative synthesis. An overall summary of recommendations was developed using the Australian National Health and Medical Research Council body of evidence framework. This framework has five components (evidence base, consistency, clinical impact, generalizability, and applicability) and an overall body of recommendation.^11^

## RESULTS

### Search Result

**Fig 1:**
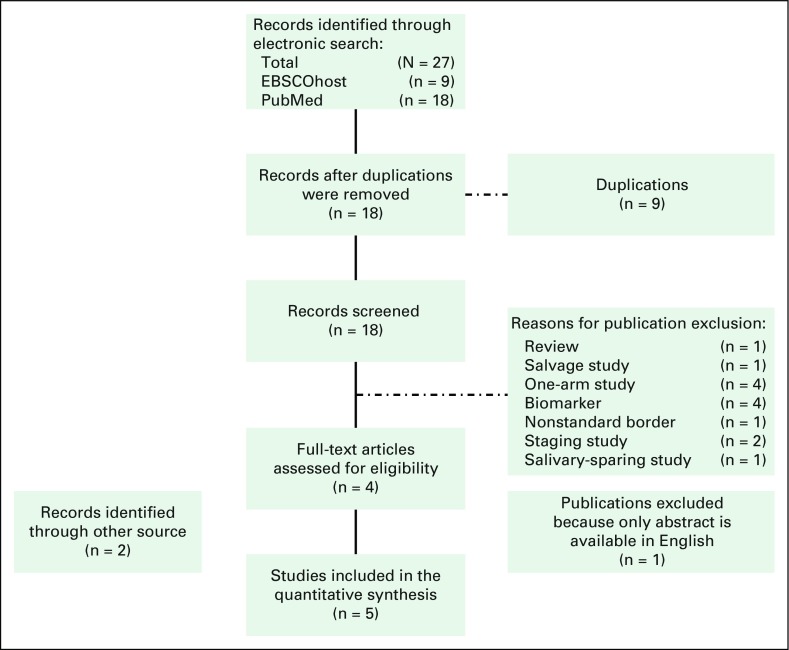
– Preferred Reporting Items for Systematic Reviews and Meta-Analyses (PRISMA).

This review initially yielded 27 abstracts (Fig 1). Twenty-three studies were excluded for the following reasons: duplication (n = 9), review study (n = 1), salvage study (n = 1), one-arm treatment (n = 4), biomarkers (n = 4), nonstandard border (n = 1), staging study (n = 2), and salivary-sparing study (n = 1). An additional two studies were included from the search of the reference lists of the included studies. Full texts of the six studies were then reviewed for eligibility. Of the six studies, one study was excluded because only the abstract form was available in English and thus critical appraisal of the methods would not be possible.^12^ A total of five trials were included in the qualitative analysis.^13-17^

### Critical Appraisal

The five studies included in this review were of sound methodologic quality (Table 1). All studies had a clear purpose, relevant background, and justification for conducting the study. One study was a randomized controlled trial,^13^ and the other four were retrospective two-arm studies.^14-17^ The randomized controlled trial had an adequate sample, and contamination and co-interventions were controlled, which would be difficult in a retrospective study. All studies had measurable outcomes with clinically significant results. All of the studies reported on dropout rates, but only three studies provided the reasons for dropout.

**Table 1:**
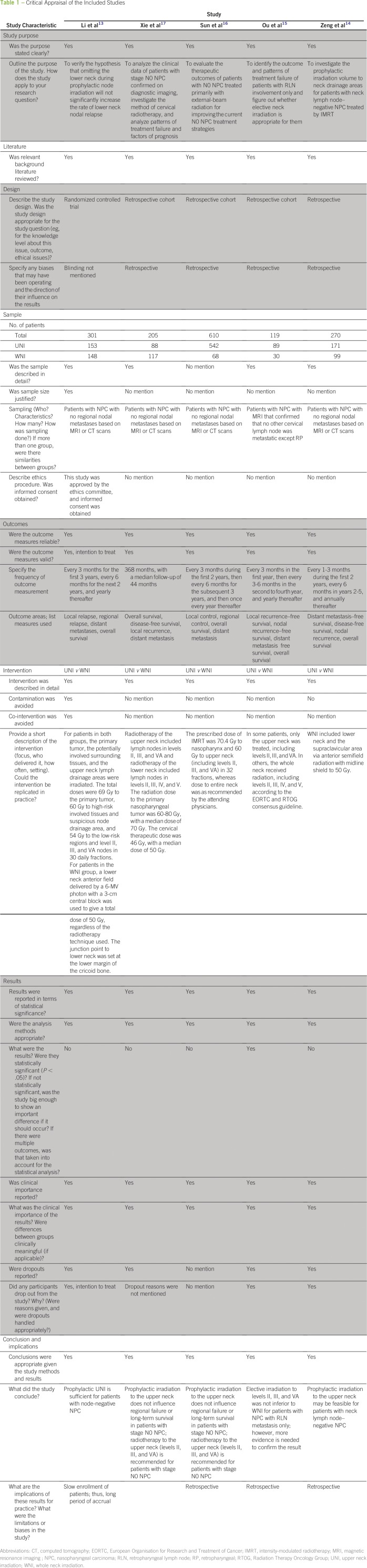
Critical Appraisal of the Included Studies

### Main Result

#### Data from randomized controlled trial.

Only one randomized controlled trial investigated the use of prophylactic UNI versus WNI in node-negative disease.^13^ The study randomly assigned node-negative patients using the sixth edition of AJCC/UICC staging system to either UNI, including levels II, III, and VA with 54 Gy, or WNI, with the addition of 50 Gy to the low anterior neck field. One hundred forty-eight patients were randomly assigned to UNI, whereas 153 patients were assigned to WNI, with a median follow-up time of 39 months. There were no confirmed nodal relapses in either arm, but two patients had suspicious nodes, one of 5 mm in the UNI arm and one of 4 mm in the WNI arm. The former occurred in level II, whereas the latter occurred in the supraclavicular fossa. The latter patient died of distant metastasis. The rates for 3-year overall survival and metastasis-free survival in the UNI versus WNI arms were 89.5% *v* 87.4% and 91.7% *v* 90.9%, respectively.

#### Pooled data from retrospective studies.

**Fig 2:**
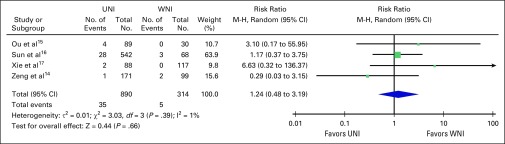
– Nodal relapse in node-negative nasopharyngeal cancer using upper neck irradiation (UNI) versus whole neck irradiation (WNI). M-H, Mantel-Haenszel test.

**Fig 3:**
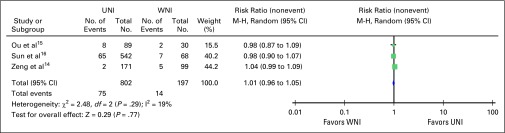
– Five-year distant metastasis–free survival in node-negative nasopharyngeal cancer using upper neck irradiation (UNI) versus whole neck irradiation (WNI). M-H, Mantel-Haenszel test.

**Fig 4:**
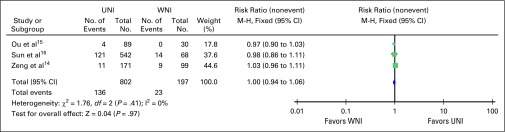
– Five-year overall survival in node-negative nasopharyngeal cancer using upper neck irradiation (UNI) versus whole neck irradiation (WNI). M-H, Mantel-Haenszel test.

Pooled analysis on nodal recurrence, whether in-field or out-of-field recurrence, showed no significant difference when UNI versus WNI was used (risk ratio, 1.24; 95% CI, 0.48 to 3.19; Fig 2). Only three studies reported on the rates of 5-year overall survival and distant metastasis–free survival; they showed no significant difference in risk of developing distant metastasis and overall survival at 5 years with risk ratios of 1.01 (95% CI, 0.96 to 1.05; Fig 3) and 1.00 (95% CI, 0.94 to 1.06; Fig 4), respectively.

## DISCUSSION

This review presented the overall evidence regarding the use of UNI versus WNI as ENI in patients with node-negative or limited RP node–positive NPC. We showed that there is no benefit of using WNI over UNI in terms of nodal relapse, distant metastasis–free survival, and overall survival. On the basis of the National Health and Medical Research Council’s “additional levels of evidence and grades for recommendations for developers of guidelines” document^11^ (Table 2), we recommend that the findings in this review regarding nodal relapse, distant metastasis, and overall survival can be trusted to guide clinical decision in most situations.

**Table 2:**
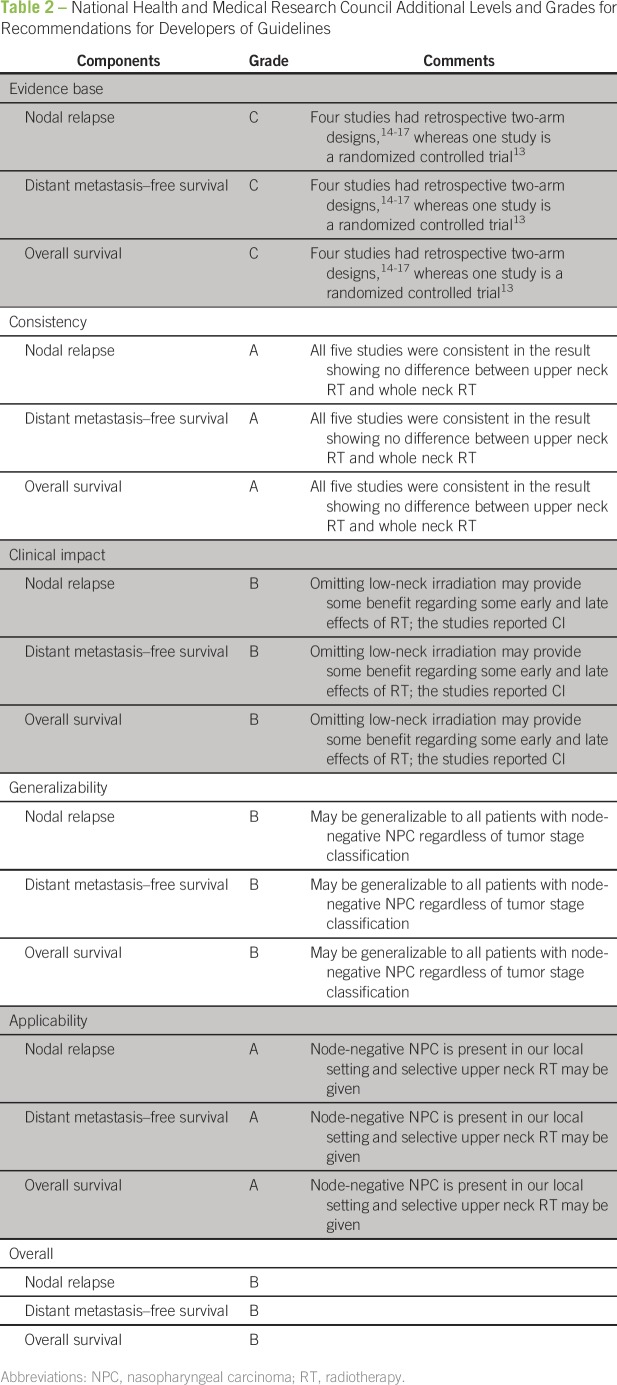
National Health and Medical Research Council Additional Levels and Grades for Recommendations for Developers of Guidelines

NPC is a common malignancy seen in southern China and Southeast Asian regions. In the Philippines, recent study showed that the incidence may still be under-reported, and the estimated incidence is approximately 2.3 per 100,000.^18^ NPC has a predilection for lymphatic metastasis as a result of its extensive avalvular submucosal cervical lymph drainage. There is orderly progression of lymph node metastasis in NPC, with the RP node being the most common, followed by levels II, III, V, and VI and the supraclavicular fossa, with only a 0.5% chance of skip metastasis.^7^ Approximately 85% of patients with NPC present with clinical cervical lymph node involvement, but a minority of patients will have node-negative disease at presentation.^7^ Although lymphatic metastasis is correlated with a higher chance of distant metastasis,^19^ it may be reasonable and logical to treat node-negative NPC with limited neck irradiation and withhold low neck radiation. A retrospective study showed that 40% of patients had nodal relapse among patients with node-negative NPC staged using clinical palpation when elective nodal irradiation was omitted. Most of these nodal relapses are successfully treated with salvage therapy, but high rates of distant metastasis (> 20%) affect overall survival.^20^ However, the results of the study by Lee et al^20^ are currently limited because of a lack of CT or MRI nodal staging in this study as well as no prophylactic irradiation in the upper neck, which in our review is a standard for all patients. A recent one-arm trial reported excellent 5-year overall survival (85%) in patients with neck-negative NPC treated with limited upper neck radiation.^21^ This is in consonance with the results of this review, in which omission of lower neck radiotherapy did not affect nodal relapse, distant metastasis, or overall survival. Because there is an orderly progression of lymph node metastasis, one or two nodal levels beyond the involved node may be sufficient for ENI. Nodal relapse may occur in the lower neck if there is insufficient coverage to this area, which may harbor microscopic disease. In three included studies, five of seven nodal relapses in the UNI arm occurred at in-field sites,^14,15,17^ whereas another study reported only 16 patients (2.7%) of the total cohort with relapse in the elective nodal area; the study also reported relapse in 13 patients (2.1%) in the out-of-field area, and relapse in two patients (6.5%) in both the in-field and out-of-field areas.^16^ The results show that coverage was adequate with UNI because treatment failures occurred mostly at in-field sites.

Although most of the structures in the neck receive tolerated doses in WNI, it is prudent to limit the dose to this area if oncologic outcomes are not compromised. A recent study showed a dose-response curve demonstrating an increased chance of development of hypothyroidism.^22^ Furthermore, limiting dose to this area may prevent intima media thickening,^23^ esophagitis, and pulmonary apical fibrosis in this subset of patients. In the studies included in this review, two studies showed no significant difference in the rates of acute and late effects, except more patients had lower neck dermatitis, skin atrophy, and lung apex fibrosis in the WNI group.^13,14^ One study reported that three patients had cranial neuropathy and one patient had unilateral laryngeal nerve palsy in the WNI group.^15^

With the advent of modern imaging, the staging of NPC has evolved from mere clinical palpation to image-guided staging. CT scan is not able to predict and differentiate primary tumor from RP nodes; the advantage of MRI is that it can delineate these nodes specifically. RP lymph node involvement may be present in 86.4% of patients when staged using MRI.^21^ The presence of positive RP nodes has prognostic implications. In the sixth edition of the AJCC/UICC staging system, the presence of positive RP nodes was staged as N0, but most studies have shown that a positive RP node behaves like N1 disease. For this reason, in the seventh edition of the AJCC/UICC staging system, disease with positive RP node was considered N1 disease. All of the studies included in this review were staged according to the sixth edition staging classification. As such, it is possible that some of the patients may have been RP node–positive. This further supports our hypothesis that radiation to one or two lymph nodes beyond the affected echelon may be adequate as ENI.

The use of cisplatin-based chemotherapy has been a standard in locally advanced NPC as concurrent treatment with radiation with or without neoadjuvant or adjuvant chemotherapy. Cisplatin has been shown to increase local control and overall survival.^3,4^ Only 33% of patients included in the review (n = 509) received some form of chemotherapy as concurrent, neoadjuvant, or adjuvant therapy. Given that only 33% of patients received chemotherapy, in the absence of subset analysis, it may be reasonable to conclude that chemotherapy did not compensate for the lack of WNI therapy to address possible microscopic disease. The 5-year survival rates of the patients, regardless of whether or not chemotherapy was given, approached the survival rates of landmark trials. This could be because high tumor stage is related to a local problem, whereas distant metastasis is related to nodal burden.

A major limitation of this review is that most of the studies are retrospective. This reflects the fact that there is a low incidence of node-negative NPC compared with node-positive NPC, as well as the lack of randomized controlled trials in node-negative NPC. Another limitation is the heterogeneity of the patients; both node-negative and RP node–positive patients were included, and the latter are considered to have N1 disease in the latest staging system. Because of the scarcity of data from randomized controlled trials, there is a need for more randomized clinical trials; these trials should stratify between N0 and RP node–positive disease. There is also a need to include late effects, such as neck fibrosis and thyroid function, as secondary outcomes. It must be noted that one study was excluded as a result of the lack of an English version of the full text, which prevented the reviewer from adequate critical appraisal of the article.^12^ Although excluded, the results of this study paralleled those of the five included studies and would not affect the overall conclusion of the review if included in the analysis.

In conclusion, in node-negative or limited RP node–positive NPC, the current evidence demonstrates the possibility of treating only the upper neck (levels II, III, and VA) without compromising nodal control, distant metastasis, and overall survival. Because of a lack of data, more randomized controlled trials are warranted in this subset of patients.
